# Chitosan-Coated Alginate Microcapsules of a Full-Spectrum Cannabis Extract: Characterization, Long-Term Stability and In Vitro Bioaccessibility

**DOI:** 10.3390/pharmaceutics15030859

**Published:** 2023-03-07

**Authors:** Aitor Villate, Markel San Nicolas, Maitane Olivares, Oier Aizpurua-Olaizola, Aresatz Usobiaga

**Affiliations:** 1 Department of Analytical Chemistry, Faculty of Science and Technology, University of the Basque Country (UPV/EHU), 48940 Leioa, Basque Country, Spain; 2 Research Centre for Experimental Marine Biology and Biotechnology (PIE), University of the Basque Country (UPV/EHU), 48620 Plentzia, Basque Country, Spain; 3 Sovereign Fields S.L., Larramendi Kalea 3, 20006 Donostia, Basque Country, Spain

**Keywords:** microencapsulation, medicinal cannabis, cannabinoids, SEM characterization, storage stability, in vitro drug release

## Abstract

Cannabinoids present in *Cannabis sativa* are increasingly used in medicine due to their therapeutic potential. Moreover, the synergistic interaction between different cannabinoids and other plant constituents has led to the development of full-spectrum formulations for therapeutic treatments. In this work, the microencapsulation of a full-spectrum extract via vibration microencapsulation nozzle technique using chitosan-coated alginate is proposed to obtain an edible pharmaceutical-grade product. The suitability of microcapsules was assessed by their physicochemical characterization, long-term stability in three different storage conditions and in vitro gastrointestinal release. The synthetized microcapsules contained mainly ∆9-tetrahydrocannabinol (THC)-type and cannabinol (CBN)-type cannabinoids and had a mean size of 460 ± 260 µm and a mean sphericity of 0.5 ± 0.3. The stability assays revealed that capsules should be stored only at 4 °C in darkness to maintain their cannabinoid profile. In addition, based on the in vitro experiments, a fast intestinal release of cannabinoids ensures a medium–high bioaccessibility (57–77%) of therapeutically relevant compounds. The full characterization of microcapsules indicates that they could be used for the design of further full-spectrum cannabis oral formulations.

## 1. Introduction

The free use of Cannabis for therapeutic purposes in 64 countries worldwide has led to a significant increase in the production of medicinal cannabis in the last 10 years [1]. Due to the proven benefits of cannabis and cannabis-based products for palliative care and the treatment of spasticity and pain [2], many patients use medicinal-cannabis-based products [3,4] to treat chronic pain and some mental illnesses instead of typical prescription drugs, such as opioids, anxiolytics and antidepressants [5].

The C_21_ terpenophenolic compounds present in the plant species *Cannabis sativa*, known as cannabinoids, are the main components responsible for the therapeutic strength of the plant, as these cannabinoids have a strong affinity for the CB_1_ and CB_2_ receptors of the endocannabinoid system [6,7]. The predominant cannabinoids in most *Cannabis* phenotypes are ∆^9^-tetrahydrocannabinol (THC) and cannabidiol (CBD). Both cannabinoids have wide-ranging medicinal applications: THC produces the psychotropic effect associated with cannabis and also has analgesic, anti-inflammatory and antiemetic properties; whereas CBD is nonpsychotropic and has anxiolytic, antipsychotic, anti-inflammatory, analgesic and anticonvulsing properties among others [7,8,9]. There are some other cannabinoids with known potential therapeutic applications present in Cannabis phenotypes, such as cannabigerol (CBG), cannabichromene (CBC), cannabinol (CBN), cannabidivarin (CBDV) and tetrahydrocannabivarin (THCV) [10]. The molecular structure of those cannabinoids and their acidic precursors, together with some basic chemical information, is summarized in Table S1.

Cannabinoids can be administrated through inhalation oral, intravenous, transdermal or rectal routes. In therapeutic applications, oral administration is the most common administration mode due to its ease of dosing, reduced toxicity and long-lasting effects, but the bioavailability of cannabinoids is lower in comparison to the rest of the administration modes [11,12,13]. However, this disadvantage depends on the edible’s matrix, so it can be overcome if cannabinoids are encapsulated in emulsions, liposomes or polymeric particles [14,15,16]. Some edible cannabinoid capsules available in the market, such as the well-known Marinol^®^ (containing dronabinol or synthetic THC) and Cesamet^TM^ (containing nabilone, a synthetic cannabinoid similar to THC), as well as some other pure THC-, pure CBD- or THC/CBD-based formulations encapsulated in different matrixes, are in advanced phases of clinical studies [9,16]. Even though the efficiency of the mentioned products is assured, the development of new products is focused on the development of products known as full-spectrum formulations. These products contain cannabinoids and other main components of the plant as well, with the aim of improving the analgesic and anti-inflammatory properties of cannabis and reducing some adverse effects that THC can cause [8,16,17].

There are few works in the literature dealing with the encapsulation of full-spectrum cannabis extracts, such as the polycaprolactone (PCL) microdepots prepared by Uziel et al. [18] and the various poly(thioether-ester) (PTEe) nanocapsules synthetized by Freire et al. [19]. Although these formulations have shown outstanding physicochemical properties, high therapeutic efficacy [18] and promising efficacy results as antitumoral agents [19], they may not be consumed orally, so further research is required to design edible full-spectrum formulations. The encapsulation of hydrophobic drugs, such as cannabinoids, is commonly achieved via emulsions due to their ease of production at large scale and their ability to be included in foods and beverages. However, the controlled release of bioactive compounds is hindered using emulsions [14,15]. Alternatively, microgels allow the controlled release of bioactive compounds in the presence of a specific stimuli (e.g., pH, temperature, chemical and biological conditions) [14,15]. Alginate, a naturally occurring polysaccharide with different polymeric structures at different pH conditions (i.e., it shrinks at low pH and swells at high pH), is widely used for encapsulation purposes due to its low cost and biocompatibility [14,20,21,22] and its ability to design microgels that allow intestinal delivery of bioactive compounds [21,23]. However, the addition of other polymers, such as chitosan, agarose, zein, poly-L-lysine or polyethyleneimine, is often required to obtain microgels with enhanced characteristics or release properties [21,22]. In this regard, chitosan-coated alginate microgels are gaining interest since the presence of chitosan reduces the pore size of the alginate network, improving thereby the storage stability of the entrapped compounds [24], their stability through the digestive tract and slowing down their release in the intestinal phase [21,22].

Microgels are often created using modified extrusion-based techniques, which allow a continuous creation of micro-sized capsules using mild conditions and nontoxic solvents [21,25]. Among them, the vibration nozzle microencapsulation (VNM) technique has been extensively used to create customizable and homogeneous particles of alginate microgels [21,26]. This technique is based on the vibration of a piezoelectric nozzle to create micro-sized alginate droplets from a continuous laminar jet of an alginate aqueous solution. These droplets are then hardened in a gelling bath solution that contains calcium chloride [26].

In this regard, this study aimed to encapsulate a full-spectrum cannabis extract using chitosan-coated alginate microcapsules via VNM. The synthetized microcapsules were characterized in terms of (i) physicochemical parameters, (ii) stability under light and temperature conditions and (iii) compound gastrointestinal release.

## 2. Materials and Methods

### 2.1. Materials and Standards

Alginic acid sodium salt from brown algae (low viscosity), chitosan (medium molecular weight), pepsin from porcine gastric mucosa (powder, ≥250 units/mg solid, lot result: 444 U/mg), pancreatin from porcine pancreas (powder, suitable for cell culture, 4 × USP specifications), bile salts (for microbiology) and sodium bicarbonate (ACS reagent, ≥99.7%) were purchased from Sigma-Aldrich Chemie GmbH (Schnelldorf, Germany). Phosphatidylcholine (Phospholipon^®^ 90 G, ≥94%) was produced by Phospholipid GmbH (Cologne, Germany). Calcium chloride dihydrate (CaCl_2_, for analysis EMSURE^®^), tri-sodium citrate dihydrate (for analysis EMSURE^®^), acetic acid (glacial 100%, anhydrous for analysis, EMSURE^®^) and hydrochloric acid (HCl, 36%) were purchased from Merck KGaA (Darmstadt, Germany). Ethanol (99.5%) was obtained from Panreac Química S.L.U. (Barcelona, Spain), and methanol (UHPLC-MS grade) and water (UHPLC-MS grade) from Sharlab (Sentmenat, Spain). Milli-Q quality water (<0.05 µS cm^−1^) was produced using a Millipore 185 from Millipore (Burlington, MA, USA).

The solutions of individual standards of cannabinoids (∆^9^-tetrahydrocannabinolic acid-A (THCA, 1000 µg/mL in acetonitrile), cannabichromene (CBC, 1000 µg/mL in methanol), cannabichromenic acid (CBCA, 1000 µg/mL in acetonitrile), cannabidiol (CBD, 1000 µg/mL in methanol), cannabidiolic acid (CBDA, 1000 µg/mL in acetonitrile), cannabigerol (CBG, 1000 µg/mL in methanol), cannabigerolic acid (CBGA, 1000 µg/mL in acetonitrile), cannabinol (CBN, 1000 µg/mL in methanol) and cannabinolic acid (CBNA, 1000 µg/mL in methanol)) were purchased from Dr. Ehrenstofer GmbH (Augsburg, Germany). The deuterated analogue ∆^9^-tetrahydrocannabinol (THC, 1000 µg/mL in methanol) was supplied by Merck KGaA (Darmstadt, Germany), and phenantrene used as an internal standard (IS) was purchased from Sigma-Aldrich Chimie (Saint-Quentin-Fallavier). A mixed fresh stock solution containing 100 μg/mL of all target compounds was prepared monthly in methanol, whereas intermediate dilutions were prepared daily according to the experimentation for the preparation of calibration stock solutions.

### 2.2. Cannabis Raw Extract

The cannabis extract used as a cannabinoid source for the preparation of microcapsules was donated from Fundación Renovatio (Donostia, Basque Country, Spain). The extract was prepared in their facilities prior to our study, where raw cannabis plant material was extracted with pure ethanol, filtrated by gravity and heated (80 °C) to remove excess ethanol. Since the cannabinoid composition of the raw cannabis extract was unknown, it was determined in our laboratories by treating 0.1 g of the extract with 5 mL of methanol in an ultrasonic bath for 15 min, after which the solution was centrifuged for 5 min at 10,000× *g*. The supernatant solution was 1:100 diluted with an IS methanol solution and analyzed using the same HPLC-DAD method described in Section 2.4.

### 2.3. Preparation of Alginate–Chitosan Microcapsules

Cannabinoid-containing alginate–chitosan capsules were prepared via VNM following a previously optimized method for microencapsulation of polyphenols [24] and the patent US8808734B2 [27] with some modifications. Briefly, 2 g of cannabis extract dissolved in 10 mL of ethanol was mixed with 50 mL of ethanol containing 30 g of phosphatidylcholine. This solution was mixed with 940 mL of a 1.6% (*w/w*) sodium alginate solution in Milli-Q water, obtaining the microencapsulation agent solution. The microcapsules were created in an encapsulator Buchi B-390 (Flawil, Switzerland) operated at a frequency of 500 Hz, voltage of 600 V, pressure of 120–130 mbar and a nozzle of 300 µm, which was selected based on the used alginate solution density. A 0.2 M CaCl_2_, 1% acetic acid and 0.05% chitosan (*w/w*) aqueous solution (pH = 2.8) were placed under the broke-up laminar jet produced by the vibration nozzle to harden the alginate droplets. The agglomerates and threaded particles occasionally formed during this step were carefully removed with a spatula from the mixture. Once alginate–chitosan microcapsules were hardened, the solution was vacuum-filtered using 10–12 µm cellulose filters, and the wet microcapsules were dried in a Coolvacuum Lyomicron freeze-dryer (Barcelona, Spain) at −60 °C and 0.037 mbar for 48 h. The obtained dry capsules were weighed and stored at −20 °C until further use.

### 2.4. Cannabinoid Content of Alginate–Chitosan Microcapsules

The cannabinoid content in dry capsules was determined by means of an HPLC-DAD-based method. First, in order to destabilize the calcium alginate gel structure and favor the release of cannabinoids, 0.1 g of dry capsules was dissolved in 1.5 mL of 0.2 M sodium citrate solution and sonicated in an ultrasonic bath for 1 h [28]. After the disruption of the capsule, the cannabinoids were extracted with 20 mL of methanol, which allowed for the precipitation of the polymeric material residues. The extract was diluted 1:4 with an IS methanol solution, resulting in 10 µg/mL of IS. Diluted aliquots were filtered with 0.22 µm polypropylene syringe disks prior to HPLC-DAD analysis and stored at −20 °C until analysis.

The quantification of the cannabinoids was executed using an Infinity 1260 LC System (HPLC) coupled to an Infinity 1260 Diode Array Detector WR (DAD), both from Agilent Technologies (Santa Clara, CA, USA). Aliquots of 5 µL were injected in a Kinetex C18 column (150 × 3 mm, 2.6 µm) with a Security Guard Ultra C18 precolumn (2 × 3 mm), both purchased by Phenomenex (Torrance, CA, USA). The separation of cannabinoids was achieved using a gradient method with mobile phases A (Water, 0.1% acetic acid) and B (Methanol, 0.1% acetic acid) at a constant flow of 0.7 mL/min. The gradient method started at 30% A and was maintained for 3 min; then, it was decreased first to 20% in 6 min and then to 5% in 3 min, which was maintained for 3 min. A was increased to 30% in 5 min and maintained for another 4 min to reach initial conditions before the next chromatographic run, which lasted 24 min in total. Cannabinoids were detected using DAD and they were quantified at 230 nm using an external calibration curve prepared in the range of 0.1 and 25 µg/mL for all target compounds (THC, THCA, CBC, CBCA, CBD, CBDA, CBG, CBGA, CBN and CBNA).

### 2.5. Encapsulation Efficiency

The encapsulation efficiency (EE%) for each cannabinoid was determined by comparing the mg of cannabinoids present after encapsulation (mg_cap_) with the total amount of each cannabinoid in the cannabis raw extract (mg_ext_), using Equation (1):

(1) $$\mathrm{EE}\;\%=\frac{{\mathrm{mg}}_{\mathrm{cap}}}{{\mathrm{mg}}_{\mathrm{ext}}}\cdot 100=\frac{m_{\mathrm{cap}}\cdot C_{\mathrm{cap}}}{m_{\mathrm{ext}}\cdot C_{\mathrm{ext}}}\cdot 100$$

where m_cap_ is the obtained mass (g) of capsules, C_cap_ is the concentration (mg/g) of each cannabinoid in capsules, m_ext_ is the employed mass (g) of cannabis extract and C_ext_ is the concentration (mg/g) of each cannabinoid in the cannabis extract.

### 2.6. Physical Characterization of Alginate–Chitosan Microcapsules

The zeta potential of dry alginate–chitosan microcapsules was measured to confirm that the chitosan coat was created successfully. To this end, a 10 mg/L aqueous dispersion was prepared and measured in triplicate with a Zetasizer Nano series from Malvern Instruments (Malvern, UK) and using a disposable zeta-potential cuvette. Measurements were obtained at an angle of 90°.

Regarding particle size and morphology of the synthetized microcapsules, the average values of those parameters were determined by means of Scanning Electron Microscopy (SEM). Dry capsules were covered with 15 nm of gold in a K550X sputter coater from Emitech (Montigny-le-Bretonneux, France) and were measured using a FEG SEM S4800 from Hitachi (Tokyo, Japan) with a 5 kV acceleration voltage. The SEM images were processed and analyzed using ImageJ software from GitHub Inc. (San Francisco, CA, USA) as described by Mazzoli and Favoni [29].

### 2.7. Long-Term Stability of Cannabinoids in Alginate–Chitosan Microcapsules

The stability of cannabinoids in alginate–chitosan microcapsules was evaluated in different temperatures and light storage conditions for 10 months (i.e., 310 days). The studied storage conditions were the following: (i) room temperature (RT) with natural day–night cycle light exposure, (ii) RT without light exposure and (iii) 4 °C without light exposure.

The long-term stability assays were designed based on the fact that cannabinoids remain stable at −20 °C for at least 4 years [30,31]. To minimize interday variability and possible surface-core differences, 0.1 g of stable microcapsules (i.e., maintained at −20 °C) was placed in three clear glass closed vials and exposed to the three different conditions tested every 15 days during the experiment, resulting in 21 sampling days per storage condition. Three experimental replicates were prepared in four sampling days (i.e., 1, 9, 15 and 21) throughout the experiment (i.e., corresponding to days 0, 121, 218 and 310 of the whole experiment, respectively) to determine the experiment repeatability. The average experiment repeatability was used to calculate the uncertainty of the cannabinoids’ concentration for the rest of the experimental sampling days. Once the period of 10 months ended (i.e., sampling day 21 after 310 days), all samples were analyzed in three batches (one per storage condition) following the method described in Section 2.4.

The quantitative comparison of degradation profiles for each cannabinoid at different tested conditions was achieved through the calculation of degradation rates constants (λ) and elimination half-life times (t_1/2_), based on a first-order reaction kinetic model:

(2) $$\ln\left(\frac{C_{t}}{C_{0}}\right)=-\lambda \cdot t$$

(3) $$t_{1/2}=\frac{\;\ln2}{\lambda }$$

where C_t_ is the concentration of each cannabinoid (mg/g) in capsules in each of the timings, C_0_ is the initial concentration of each cannabinoid (mg/g) in capsules and t is the corresponding storage time expressed in days.

### 2.8. In Vitro Gastrointestinal Release

The release of the encapsulated cannabinoids was determined by a static in vitro simulation of the gastrointestinal digestion, following the recommendations of INFOGEST [32] and Minekus et al. [33]. Simulated gastric fluid (SGF) contained 2000 U/mL of pepsin and 0.17 mM of phosphatidylcholine in Milli-Q water, and the pH was adjusted to 3 with HCl 36% solution. Simulated intestinal fluid (SIF) contained 3.2 g/L of pepsin and 8.16 g/L of bile salts in Milli-Q water.

For the bioaccessibility assays, 0.3 g of dry alginate–chitosan microcapsules was placed in 10 mL of SGF at 37 °C under continuous stirring for 2 h to simulate the gastric phase. After that, 10 mL of SIF was added (resulting in 1.6 g/L of pepsin and 4.08 g/L of bile salts), pH was adjusted to 7 using a saturated sodium bicarbonate aqueous solution and the mixture was maintained at 37 °C under continuous stirring for 4 h, simulating the intestinal phase. The release profile of cannabinoids was determined by taking aliquots of 200 µL at different timings (12 aliquots during the 6 h of the experiment). After each aliquot was taken, 200 µL of fresh SGF or SGF:SIF 1:1 mixture was added to the assay solution in order to maintain the microcapsules–simulated fluids ratio. The entire simulation was conducted in triplicate.

All the aliquots were diluted 1:2 using 20 µg/mL IS methanol solution and centrifuged with a 5424 R Eppendorf Centrifuge (Hamburg, Germany) for 5 min at 10,000× *g*. The supernatants were collected and filtered with 0.22 µm polypropylene syringe disks prior to HPLC-DAD analysis, which was performed as explained in Section 2.4.

The results were expressed as the cumulative fraction of released cannabinoids from the total cannabinoid content through digestion time. Intestinal bioavailability was calculated by resting the fraction released in the gastric phase to the final released fraction.

## 3. Results and Discussion

### 3.1. Cannabinoid Content and Encapsulation Efficiency

The concentration of cannabinoids in the raw extract used for the encapsulation and in the microcapsules and the encapsulation efficiency are displayed in Table 1.

The profile of cannabinoids in the raw extract is dominated by the presence of THC-type (THC + THCA) and CBN-type (CBN + CBNA) cannabinoids with overall concentrations higher than the rest of the cannabinoids. The relatively high THC-type cannabinoids content compared to the low CBD-type (CBD + CBDA) content indicates that the extract used in microencapsulation assays belonged to a Chemotype I (or THC-rich) plant [34]. In addition to this, the concentration of CBN-type cannabinoids is unusually high. CBN and CBNA are the two main oxidation degradation products of THC and THCA and are normally encountered in the levels of minor cannabinoids in fresh plant material [10,35]. This observation suggests that the raw extract was probably stored at RT for a large period of time prior to its reception [36]. Anyhow, the concentration of cannabinoids present in the raw extract was enough to perform the microencapsulation.

The microencapsulation protocol described in this work rendered adequate encapsulation efficiencies between 86% and 104%, except for CBDA. These results indicate that no significant loss of cannabinoids occurs during the microencapsulation process. The lower encapsulation efficiency calculated for CBDA (i.e., 75%) could be attributed to its very low concentration in the raw extract. The obtained encapsulation efficiencies are comparable to those obtained in previous full-spectrum encapsulation studies. Uziel et al., for example, obtained encapsulation efficiencies between 90% and 102% in their PCL microdepots [18], and, similarly, Freire et al. obtained encapsulation efficiencies above 97% in all the different PTEe nanoparticles they synthetized [19].

Considering the overall cannabinoid concentration in the obtained microcapsules, they could be classified as low-dosing microcapsules for therapeutic cases. Based on the literature, it is widely accepted that the effective dose of THC is close to 2.5 mg [16,37]. In fact, clinical studies have used oral doses of THC ranging from 2.2 mg to 100 mg [13,14], whereas Marinol^®^ capsules are available in doses of 2.5, 5 and 10 mg of THC. Therefore, around 2 g of alginate–chitosan capsules would be enough to reach the minimum THC dose. With that dose, the effective dose of the other two major cannabinoids (i.e., CBN and THCA) might also be assured, since the oral dose of CBN in clinical studies ranges between 20 mg and 1200 mg [38], whereas the oral dose for THCA is still not established [10].

Regarding the applicability, CBN and THCA have a lower affinity with CB_1_ and CB_2_ receptors compared to THC, and they produce similar therapeutic effects with lower potency [10,35]. Interestingly, it has been believed that aged cannabis, or a combination of CBN with THC, has sleep induction or sedation properties, and products have been sold under this premise. However, no significant evidence of that effect has been found [35,38,39]. Thus, the applicability of the synthetized microcapsules could be similar to THC-predominant formulations with the possible benefits of the entourage effect of a full-spectrum formulation.

In any case, the cannabinoid profile and amount in alginate–chitosan microcapsules depend on the employed cannabis extract, so different extracts could be used in order to obtain capsules with the desired content, which seems possible based on the high encapsulation efficiencies. The profile can be easily changed by using extracts obtained from other chemotypes, and the amount can be raised by using more purified extracts. Together with this, the amount of neutral cannabinoids (i.e., THC, CBN, CBD, CBC and CBG) can be raised by the decarboxylation of acidic cannabinoids (i.e., THCA, CBNA, CBDA, CBCA and CBGA) [36]. In this regard, even though heating plant raw extracts is a common practice to produce products rich in neutral cannabinoids [37,40], acidic cannabinoids with known therapeutic or synergistic properties are lost [8,10] along with other synergistic constituents of cannabis, such as terpenes or flavonoids [40].

### 3.2. Physical Properties of Capsules: Zeta Potential, Size and Shape

Zeta-potential measurements revealed that the surface charge of capsules was 15 ± 6 mV. This positive value of zeta potential confirmed that a positively charged chitosan layer was formed successfully above the calcium alginate polymeric structure [41,42,43,44]. Zeta-potential values above 30 mV are usually sought in order to avoid particle aggregation or flocculation in aqueous dispersion [41,45,46] and as reported by Khan et al. in their study with resveratrol alginate–chitosan zein nanocapsules; this zeta potential could be increased with higher chitosan to alginate ratios [43]. Nonetheless, the obtained zeta potential was considered sufficient taking into account the particle size of the obtained alginate–chitosan microcapsules, because in micrometric-sized particles the effect of zeta potential on colloidal stability is not as critical as in nanometric-sized particles [47].

**Figure 1:**
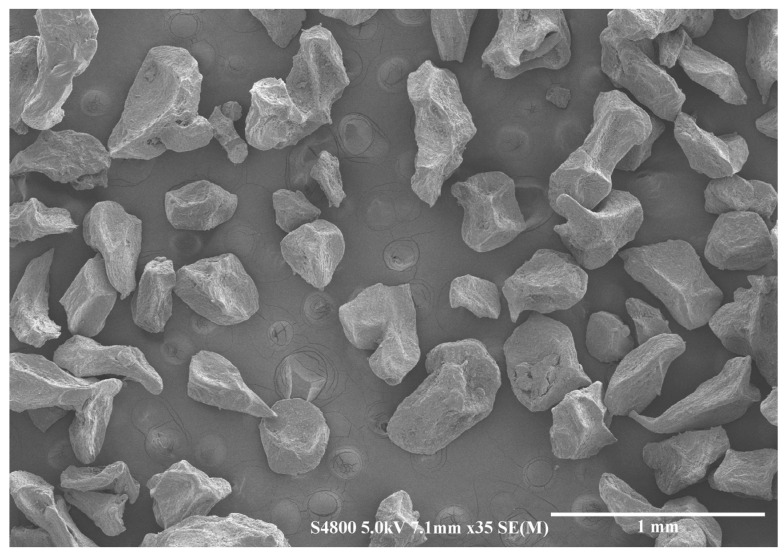
SEM image of alginate–chitosan microcapsules.

**Figure 2:**
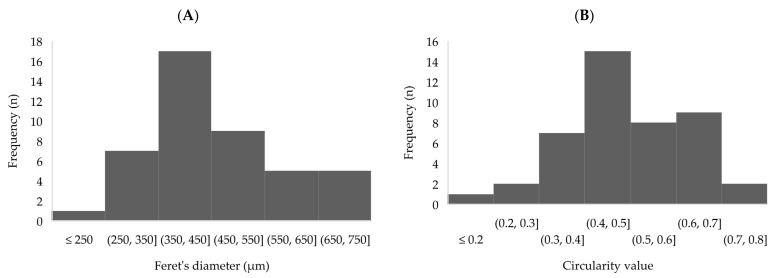
Histograms of the obtained Image J (Bethesda, MD, USA) results from Figure 1 image: (**A**) Feret’s diameters and (**B**) circularity values, where 1 refers to a perfect circle shape and values near 0 refer to elongated shapes.

Overall micrometric-sized, angular-shaped particles were obtained (Figure 1). SEM image analysis was used for the determination of Feret’s diameters (d_f_) (Figure 2A) and circularity values (Figure 2B) of the microcapsules, which were used to evaluate particle size and shape distributions, respectively.

The mean d_f_ of synthetized microcapsules was 460 ± 250 µm at a 95% of confidence level (expressed as 2 s). The measured size matches the expected size, given that a 300 µm nozzle was used, and the chitosan coating increases the particle size [43,44,48]. The observed particle size distribution (CV = 27%), which is close to a normal distribution (Figure 2A), is larger than expected for a lab-scale VNM technique (CV = 5%) [26,49], although in those cases the presence of a chitosan coat and the drying step are not considered. Nonetheless, for most encapsulation applications, a particle size between 200 and 800 µm is sought [50], and this condition is met (Figure 1 and Figure 2A).

Regarding the shape of the capsules, a circularity mean value of 0.5 ± 0.3 (at a 95% confidence level) was determined (Figure 2B), indicating that most of the microcapsules were semi-spherical, and the rest were spherical and/or microcapsules with deformations or vast elongations. The lack of sphericity in some microcapsules can cause a reduction in their mechanical and chemical resistance [50]. Many of the factors that affect particle size (i.e., alginate concentration, voltage, needle size, encapsulation flowrate and hardening solution’s surface tension, viscosity and stirring rate [26,49,50]) were previously optimized following the same methodology used in our research group [24]. Hence, the lack of complete sphericity could be attributed to the freeze-drying step, which affects negatively the sphericity [42,51].

The synthetized microcapsules present a rough and porous surface (Figure 1). Porosity can affect the retention, protection and release of the bioactive compounds [52], making it a key factor in explaining their stability and bioaccessibility in the following sections. Nevertheless, the observed porosity did not cause the loss of cannabinoids during the encapsulation process, as the encapsulation efficiencies reveal.

Comparing these results with previous full-spectrum formulations, Uziel et al. obtained 260 µm spherical PCL particles [18], whereas Freire et al. achieved nanometrically sized (100–200 nm) spherical particles in their PTEe-based formulations [19]. Such differences, however, are intrinsic to the polymeric materials and the encapsulation method or technology employed for capsule creation. Moreover, those formulations, along with other cannabinoid-based nanocapsules [53,54,55], are designed to be taken intravenously or intraperitoneally, where the particle size and shape are much more critical than the ones required for edible products. Berrocoso et al., for instance, obtained edible nanometrically sized particles, loaded with CB13 (a synthetic cannabinoid) using poly-lactic-co-glycolic acid (PLGA). This polymer and the employed nanoprecipitation encapsulation method, though, were specifically chosen to create nanocapsules capable of entering the bloodstream from the digestive tract and, thus, prolonging the release of CB13 for several days [56]. Therefore, we consider that the obtained results are satisfactory, as they are sufficient to fit with the main aim of this work.

### 3.3. Long-Term Stability of Cannabinoids

**Figure 3:**
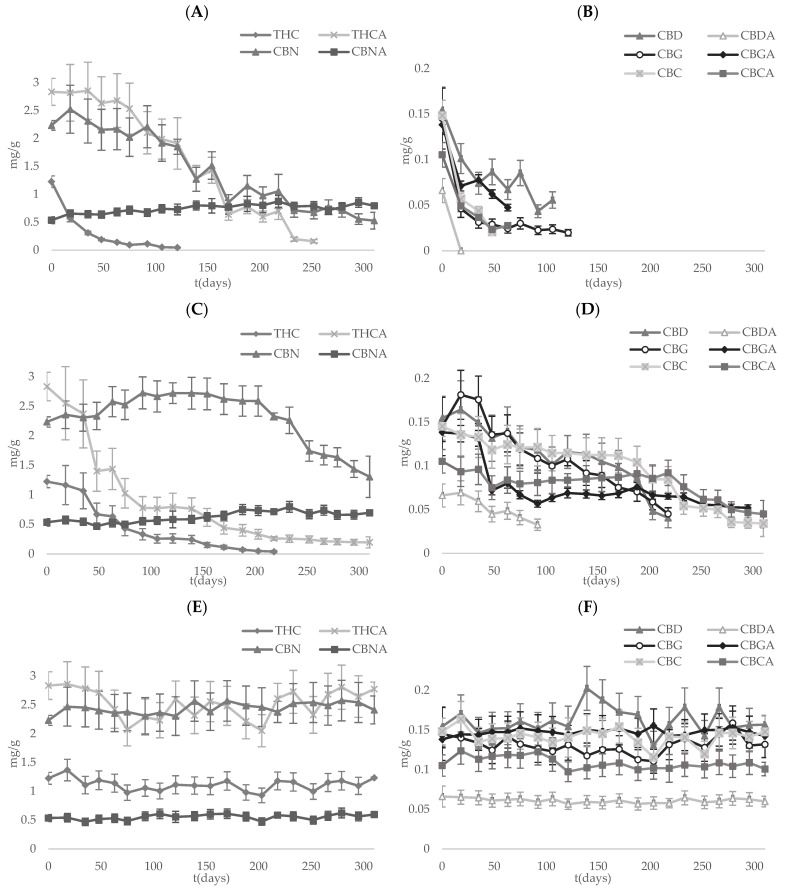
Evolution of cannabinoid content in different storage conditions: (**A**,**B**) at RT with natural day–night cycle light exposure; (**C**,**D**) at RT without light exposure; (**E**,**F**) at 4 °C without light exposure.

The concentration of cannabinoids in the capsules subjected to different light and temperature conditions for 10 months was determined, and the time profiles are shown in Figure 3. In order to determine degradation profiles quantitatively, the degradation rate constants calculated for each target compound in the different tested conditions are summarized in Table 2.

Based on the results, all cannabinoids, except CBNA, show similar degradation trends in microcapsules stored at light and room temperature conditions (see Figure 3A,B). Under these conditions, the concentration of cannabinoids decreased continuously from the beginning, reaching concentrations below the limit of detection within the first 3 months of storage. The t_1/2_ was between 2 and 5 weeks in all cases, with the exceptions of CBD (60 days), THCA (90 days), CBN (170 days) and CBNA (−394 days). The oxidative degradation of cannabinoids could be the main reason for the concentration decay [57,58], but the decarboxylation of acidic cannabinoids can also occur under these conditions [36].

Although an initial increase in the content of neutral cannabinoids could be expected [29,53,54], in this work, the degradation of neutral cannabinoids was faster than their formation (λ of neutral cannabinoids > λ of their relative acidic species). It can also be observed that CBC, THC and CBG have the biggest λ, in contrast to CBN, which has the smallest λ, probably due to the oxidation of THC to CBN [51]. Moreover, the negative degradation constant of CBNA indicates the importance of the oxidation process at those storage conditions, as the formation of CBNA, due to the oxidation of THCA [35], is faster than the degradation of CBNA.

The degradation patterns observed for the cannabinoids present in microcapsules stored at RT and darkness (Figure 3C,D) were similar to the ones observed for microcapsules stored at RT and light, but the degradation was slower. In fact, the degradation constants found for all cannabinoids were between 2 (for THC) and 10 (for CBC and CBCA) times smaller when stored in darkness at the same temperature (Table 2), except for THCA (λ = 0.0098 days^−1^). Hence, the t_1/2_ values were raised to at least 4 months in all cases except for THC (48 days) and THCA (70 days). Similarly to what was found for CBNA at RT and light storage conditions, with the absence of light, a negative degradation constant was also found, but the value was almost twice as smaller compared to the value obtained for microcapsules stored in the presence of light. The degradation pattern of CBN, with a U-inverted curve type pattern (r^2^ = 0.1155 for a first-order kinetic model), is the most significant difference between the microcapsules stored with and without light. Specifically, under darkness, the formation of CBN is favored because the oxidation of THC occurs faster than the degradation of CBN (Figure 3C), so the degradation of CBN is only visible when THC is completely oxidized (day 200).

The concentration pattern of cannabinoids encapsulated in the microcapsules stored at low temperature (4 °C) and darkness remained constant, with no statistically significant variation (*p*-value >> 0.05) during the studied period (Figure 3E,F). As a result, most of the proposed degradation kinetic model regressions showed a lack of fit, and the λ values dropped drastically.

The observed results match the cannabinoid degradation patterns in other products maintained in similar storage conditions. Zamengo et al. reported mean t_1/2_ values of ∼500 to 660 days for THC, ∼1300 to 3000 days for CBD and ∼2500 to 2600 days for CBN in marihuana and hashish samples stored at 22 °C for 24 h light exposure and in darkness, respectively [31]. Compared to our results, the t_1/2_ of cannabinoids is more than 10 times bigger in marihuana and hashish in both storage conditions. Similarly, the degradation of cannabinoids was faster compared to the observations made in long-term stability studies of plant material [59,60,61], hashish [30,59,62,63], extractions in organic solvents [30,64] and oils [65]. Temperature is the main factor that accelerates cannabinoid degradation, as it accelerates the oxidation of cannabinoids or decarboxylation of acidic cannabinoids [30,31,36,59,60,61,62,64]. This phenomenon is increased by the presence of light, as it has been previously reported [30,31,59,60,62]. Consequently, in order to conserve the initial concentration of cannabinoids, low temperatures (4 °C) and darkness are required for long-term storage periods [30,31].

The porous nature of alginate microcapsules can facilitate the entrance of oxygen molecules, which can oxidize encapsulated cannabinoids [52]. This is not so easily occurring in plant material, hashish, organic solvent extracts or oils, where cannabinoids can remain more stable [30,64,65]. In addition to this, the capsules have a larger contact surface than any solid cannabis product, and the fact that they were stored in small amounts (0.1 g) could lead to higher degradation [30,59]. This suggests that the chitosan coat may not fill enough of the pores of the alginate microgel to block the entrance of oxygen and to protect the cannabinoids from oxidation, at least at room temperature.

The results obtained in this section suggest that the chitosan-coated alginate microcapsules require the use of a refrigerator to ensure the maintenance of their cannabinoid profile. Such requirements can be quite common in cannabinoid-containing products. For example, the manufacturers of the above-mentioned Marinol ^®^ capsules recommend the storage of the product “between 8 and 15 °C or alternatively in a refrigerator” [66].

### 3.4. In Vitro Gastrointestinal Release

**Figure 4:**
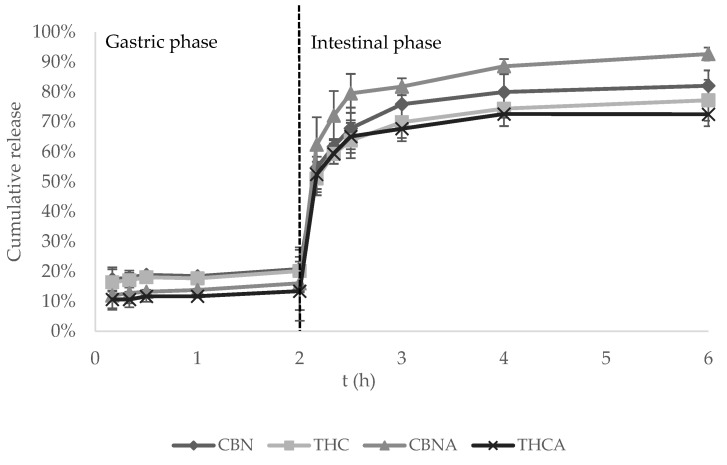
Gastrointestinal cumulative release (%) of CBN, THC, CBNA and THCA.

Figure 4 shows the cumulative cannabinoid release of main cannabinoids (expressed in % of the total content) during the gastrointestinal digestion. The evolution of the cumulative release profile of the four measured cannabinoids [52] suggests a delayed release. In the gastric phase, a soft release of 13–21% occurred in the first 2 h. Once the intestinal phase started, a burst release occurred in the first 10 min, where the cumulative release rises between 51% and 61%, followed by a sustained release until the end of the simulation, with between 73% and 93% of the total content in capsules released. This resulted in an intestinal bioaccessibility of 61 ± 9% for CBN, 57 ± 10% for THC, 77 ± 9% for CBNA and 59 ± 11% for THCA.

Comparison of these results with the observations found by Ribeiro et al., who studied the release of lipophilic drugs from coated and uncoated alginate capsules using a similar in vitro release model [67], suggests that the release of cannabinoids from the alginate–chitosan microcapsules is more similar to the release profile of uncoated alginate capsules than the release of chitosan-coated ones. In their study, the intestinal phase release of lipophilic drugs in the chitosan-coated alginate capsules was more prolonged but incomplete, with a cumulative release after 12 h that did not exceed 35% of the total encapsulated drug content. This, along with the observations of previous sections, suggests that the chitosan coating was not sufficient to cover the pores of the uncoated alginate capsules.

Burst releases are usually undesired in order to avoid toxic concentrations and short half-lives of bioactive compounds in plasma [68]. However, this kind of profile might be suitable in these low-dose microcapsules to ensure enough cannabinoid concentration in the blood to provide the sought therapeutic effects. In addition, the obtained release profile might be of high interest owing to the higher and repetitive in vitro intestinal bioaccessibility, although further pharmacokinetic experiments should be carried out to assess bioavailability.

The acidic degradation of cannabinoids in the stomach is one of the factors that contribute negatively to their oral bioavailability [12]. The observed high bioaccessibility (57–77%) suggests that the gastric degradation of encapsulated cannabinoids is negligible and that there is a high fraction to be absorbed. However, this observation together with the observed low CV % (<10%) does not necessarily result in a more repetitive or larger bioavailability. In fact, the oral absorption of cannabinoids is highly variable, even with the same edible product, and the first-pass hepatic metabolism is still a big barrier to overcome, as a high fraction of cannabinoids is usually lost in this step due to the transformation into inactive metabolites [12,69]. Therefore, although the in vitro bioaccessibility assays’ results seem promising, further experiments are needed to assess the overall bioavailability of the encapsulated cannabinoids, which generally involves carrying out in vivo experiments [70], especially taking into account the complex matrix of the alginate–chitosan microcapsules (i.e., different cannabinoids, other plant constituents present in the extract, phospholipidic micelles).

## 4. Conclusions

Full-spectrum oral cannabis formulations are becoming increasingly popular in the field of therapeutic cannabis due to the synergistic interaction between different cannabinoids and other components of the plant, as well as the convenience and safety of oral administration. In this case, a full-spectrum formulation, predominant in THC-type and CBN-type cannabinoids, has been developed, which may have an application in therapies that demand low dosing of these cannabinoids. There are a variety of encapsulating materials and methodologies that can provide optimal physicochemical properties, storage stability or digestion release properties. The obtained chitosan-coated alginate capsules via the VNM technique in this work offer standard quality properties, regarding their size and shape, the need to store them at 4 °C and their behavior in gastrointestinal media. Nonetheless, compared to other encapsulation methods and materials, our method only requires materials obtained from natural sources, which are abundant and easy to obtain, and therefore have a low cost. It does not require the use of any type of high-cost technology, include any step that may pose a potential risk or generate any harmful waste to health or the environment, as it does not require the use or production of any organic solvents, plastics or other types of pollutants. In addition, this study opens a window for a vast number of new cannabis full-spectrum formulations since any cannabis extract can be encapsulated following a customizable synthesis pathway (i.e., type of coating, drying method) in order to obtain formulations with desired properties. In any case, further bioavailability assays should be carried out to obtain more precise information about their pharmacokinetics and, thus, their suitability.

## Figures and Tables

**Table 1:** Mean concentration of cannabinoids (mg/g) in extract and microcapsules (n = 3, 2 s at 95% confidence level) and encapsulation efficiency.

Cannabinoid	C_ext_ (mg/g)	C_cap_ (mg/g)	EE (%)
THC	21 ± 1	1.2 ± 0.1	98%
THCA	49 ± 3	2.8 ± 0.2	96%
CBN	42 ± 3	2.2 ± 0.1	87%
CBNA	8.8 ± 0.6	0.53 ± 0.06	101%
CBD	3.0 ± 0.2	0.15 ± 0.02	87%
CBDA	1.5 ± 0.1	0.07 ± 0.01	75%
CBG	2.3 ± 0.3	0.14 ± 0.03	104%
CBGA	2.3 ± 0.2	0.14 ± 0.01	9 9%
CBC	2.6 ± 0.2	0.15 ± 0.02	95%
CBCA	2.0 ± 0.2	0.11 ± 0.01	86%

C_ext_: cannabinoid content in extract; C_cap_: cannabinoid content in capsules; EE (%): encapsulation efficiency.

**Table 2:** Degradation rate constants (λ) of cannabinoids at different conditions.

	RT with Light Exposure			RT without Light Exposure			4 °C without Light Exposure		
	λ	r^2^	t_1/2_	λ	r^2^	t_1/2_	λ	r^2^	t_1/2_
THC	0.0298	0.9815	23	0.0144	0.9874	48	0.0005	0.4471	1426
THCA	0.0077	0.8669	90	0.0098	0.9891	70	0.0006	0.4206	1231
CBN	0.0041	0.9365	170	0.0004	0.1155	1679	−0.0005	0.8118	−1466
CBNA	−0.0018	0.8873	−394	−0.0010	0.8209	−680	−0.0002	0.2481	−2913
CBD	0.0116	0.9313	60	0.0039	0.8540	177	−0.0002	0.1172	−3534
CBDA				0.0065	0.9302	107	0.0004	0.6986	1613
CBG	0.0206	0.8994	34	0.0038	0.8948	180	0.0005	0.4386	1426
CBGA	0.0176	0.9505	39	0.0039	0.8895	177	−0.0003	0.7005	−2170
CBC	0.0401	0.9855	17	0.0035	0.8897	196	0.0003	0.3161	2511
CBCA	0.0265	0.9552	26	0.0016	0.8016	440	0.0000	0.0039	−27,211

Degradation rate constants in days^−1^ (λ), coefficient of determination from linear regression models (r^2^) and degradation half-life values in days (t_1/2_).

## Data Availability

Not applicable.

## Supplementary Materials

The following supporting information can be downloaded at https://www.mdpi.com/article/10.3390/pharmaceutics15030859/s1, Table S1: Chemical structure, name, molecular formula, average mass and log P of the most relevant cannabinoids.
