# Supplementary material for: Chitosan-Coated Alginate Microcapsules of a Full-Spectrum Cannabis Extract: Characterization, Long-Term Stability and In Vitro Bioaccessibility
Source: Pharmaceutics. 2023 Mar 7;15(3):859. doi: 10.3390/pharmaceutics15030859 (PMC10058102; doi:10.3390/pharmaceutics15030859)
Supplement: Supplementary file 1 [file pharmaceutics-15-00859-s001.zip › pharmaceutics-2218493-supplementary.pdf]

Table S1. Chemical structure, name, molecular formula, average mass and log P of the most relevant cannabinoids.

Chemical Structure	R	Name (Abbreviation)	Molecular formula<br>Average mass (Da)<br>Log P
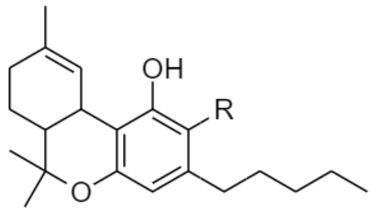	H	Δ9-tetrahydrocannabinol (THC)	$C_{21}H_{30}O_2$<br>314.462<br>7.68
	COOH	Δ9-tetrahydrocannabinolic acid (THCA)	$C_{22}H_{30}O_4$<br>358.471<br>8.41
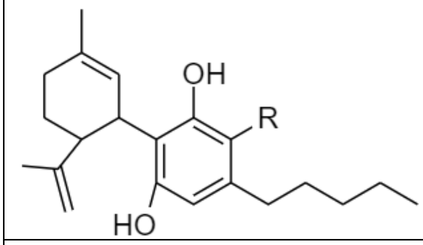	H	Cannabidiol (CBD)	$C_{21}H_{30}O_2$<br>314.462<br>7.03
	COOH	Cannabidiolic acid (CBDA)	$C_{22}H_{30}O_4$<br>358.471<br>7.87
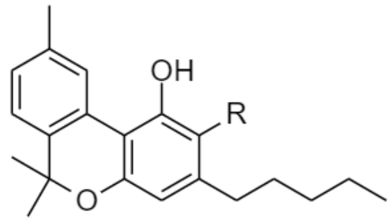	H	Cannabinol (CBN)	$C_{21}H_{26}O_2$<br>310.430<br>7.35
	COOH	Cannabinolic acid (CBNA)	$C_{22}H_{26}O_2$<br>354.439<br>8.08
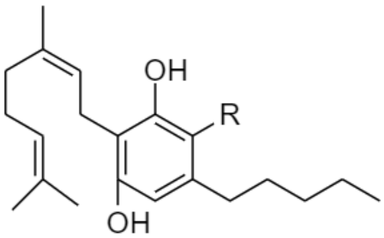	H	Cannabigerol (CBG)	$C_{21}H_{32}O_2$<br>316.478<br>7.47
	COOH	Cannabigerolic acid (CBGA)	$C_{22}H_{32}O_2$<br>360.487<br>8.31
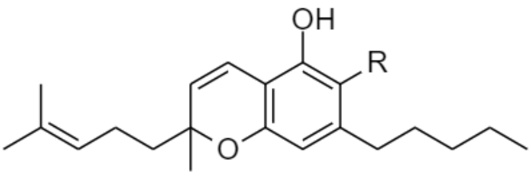	H	Cannabichromene (CBC)	$C_{21}H_{30}O_2$<br>314.462<br>8.56
	COOH	Cannabichromenic acid (CBCA)	$C_{22}H_{30}O_4$<br>358.471<br>9.29
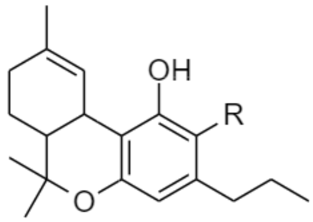	H	Tetrahydrocannabivarin (THCV)	$C_{19}H_{26}O_2$<br>286.409<br>6.62
	COOH	Tetrahydrocannabivarinic acid (THCVA)	$C_{20}H_{26}O_2$<br>330.418<br>7.34
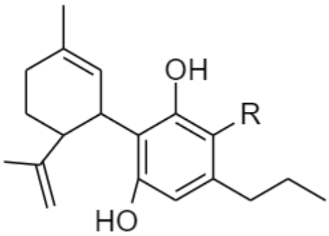	H	Cannabidivarin (CBDV)	$C_{19}H_{26}O_2$<br>286.409<br>5.97
	COOH	Cannabidivarinic acid (CBDVA)	$C_{20}H_{26}O_2$<br>330.418<br>6.81

The chemical structures were drawn in *Chemdraw* and all the shown information was extracted from *Chemspider*
